# What are the correlates of intention to be physically active in Brazilian adolescents? A network analysis

**DOI:** 10.1186/s12889-023-17291-2

**Published:** 2023-12-08

**Authors:** Cayo Vinnycius Pereira Lima, José Ywgne, Mabliny Thuany, Raphael H. O. Araujo, Ellen C. M. Silva, João C. N. Melo, Paulo Felipe R Bandeira, Leonardo G. O. Luz, Danilo R. Silva

**Affiliations:** 1 Graduation Program in Physical Education, Federal University of Sergipe (UFS), São Cristóvão, Brazil; 2 Kinanthropometry, Physical Activity and Health Promotion Laboratory (LACAPS), Federal University of Alagoas, Campus Arapiraca, Arapiraca, Brazil; 3 Department of Physical Education, Federal University of Sergipe (UFS), São Cristóvão, Brazil; 4 Centre of Research, Education, Innovation and Intervention in Sport, Faculty of Sport, Faculty of Sports, University of Porto, Porto, Portugal; 5 Graduation Program in Health Sciences, Londrina State University, Londrina, Brazil; 6 Regional University of Cariri, Crato, Ceará, Brazil; 7 Federal University of the São Francisco Valley, UNIVASF, Petrolina, Brazil; 8 The Research Center for Sport and Physical Activity (CIDAF), University of Coimbra, Coimbra, Portugal; 9 Faculty of Health Sciences, Universidad Autónoma de Chile, Providencia, Chile

**Keywords:** Adolescence, Physical activity, Physical Education, School

## Abstract

**Objective:**

The current study aimed to explore the association of individual characteristics, social and environmental factors - school and region - in the intention to be physically active in Brazilian adolescents.

**Methods:**

This is a cross sectional study based on the third edition of the National School Health Survey. The study included a total of 53,937 adolescents. To assess the intention to be physically active, only who engaged in less than 300 min of physical activity per week were included. Participants were asked: “If you had the opportunity to practice physical activity most days of the week, what would your attitude be?” Individual characteristics, physical activity domains, social factors, school, and regional environments were used as exposures. Network analysis was utilized to evaluate the associations.

**Results:**

We observed that boys had higher intentions to be physically active compared to their peers, as did adolescents who perceived themselves as fat. In addition, students from private schools show a higher intention to regularly engage in physical activities, and in general, private schools offer more extracurricular physical activities.

**Conclusion:**

In conclusion, individual factors such as sex and body image perception, and environmental factors such as school administrative dependency and availability of extracurricular activities had a significant contribution to the intention to be physically active among Brazilian adolescents.

**Supplementary Information:**

The online version contains supplementary material available at 10.1186/s12889-023-17291-2.

## Introduction

The practice of physical activity is a fundamental component of a healthy lifestyle, being one of the main issues of public health [1]. However, declines in activity levels have been observed worldwide [2–4], with more than 80% of school-aged adolescents aged 11 to 17 not meeting current recommendations for daily physical activity [5]. Data indicate that Brazil follows these trends [6]. Approximately half of Brazilian adolescents did not engage in physical activity during 2016 [6]. Furthermore, in the assessment of the Brazil Report Card 2022, the overall physical activity indicator received a grade of D, meaning that only 29.9% of children and young people met the recommendations [7]. Considering the outcomes associated with mental and physical health [8, 9] to promote opportunities for daily physical activity among adolescents remains a significant public health challenge [10]. In addition, given the moderate stability of physical activity throughout adulthood, adolescence is an important period for changing habits [11, 12], indicating the need for interventions prior to reaching adulthood.

Therefore, increasing physical activity levels is a great challenge, since physical activity is a complex behavior, influenced by a plethora of factors [13]. In addition to individual, interpersonal, political, and global factors, focusing on various psychosocial factors related to behavior, such as adolescents’ motivation for physical activity, may be a necessary step to increase their physical activity [14, 15]. Motivation is defined as something that drives action [16], which can be linked to an internal or external component [16], can promote lifelong physical activity behaviors [17], and, thus, can be considered an important correlate and a potential determinant of physical activity [18].

Studies consistently indicate lack of motivation as a major barrier to health behavior adherence and it has been suggested that motivation is one of the most under-addressed aspects to improve adherence [19, 20]. In physical activity motivation research, the Self Determination Theory (SDT) is frequently used [21]. SDT focuses on differences in the ways in which people’s behavior can be regulated and how these differences are experienced. The highest level of motivation, intrinsic motivation, arises from the willingness to understand, practice, and master a task [16], referring to participation in physical activity for fun and pleasure, while extrinsic motivation refers to external rewards or demands [22]. Overall, SDT has improved the understanding of exercise behavior, demonstrating the importance of intrinsic or autonomous regulations in fostering physical activity [23–26].

While motivation is the drive for a particular action, decision making is something more complex. The Theory of Planned Behavior (TPB) [27, 28] is one of the most widely used explanatory models in recent years to try to explain the processes that underlie decision making [29]. Based on the model, the immediate determinant of the adolescent’s exercise behavior would be the intention to perform it. For the physical exercise behavior to occur successfully, the adolescent must have a positive attitude towards physical activity, considering their beliefs, social norms, and perceived control [30]. TPB and SDT have been used by researchers trying to explain the processes that underlie motivated behavior [29].

Previous studies highlight that a positive attitude towards physical activity is the predictor variable that carries the most weight in future intentions to engage in physical activity [31, 32]. In contrast, other studies present the perception of having control and the ability to regularly engage in physical activity as the most crucial variable [33]. Añez et al. [34] also draw attention to the fact that a poor body image can act as a barrier to physical activity. Additionally, Neumark-Sztainer et al. [35] explain that adolescents with low body satisfaction are less likely to engage in physical activity, instead being more likely to spend their time in sedentary activities.

Furthermore, the social context can also function as support for adolescents to engage in physical activity [30]. Adolescents’ perceptions of their parents’ attitudes and behaviors in the area of physical activity are associated with self-perception of abilities, attitudes, and attraction to physical activities [21]. Previous studies have also highlighted the importance of students’ perceptions of physical education classes as a satisfactory experience. This will provide a range of positive consequences in their lives, such as increased psychological well-being [36] and increased intention to be physically active in the future [37], reflecting better physical and mental health.

Despite the contribution of these studies to deeply understand the association between psychosocial factors and intention to be physically active [17, 18], to understand the multifactorial nature of physical activity, a synergy between theory and methodological aspects needs to be considered. In this sense, the complex systems approach was previously applied in different settings [38]. Complex systems are open systems, in which the relationship between different variables are investigated considering the non-linearity, as well as the interplay established between the subject and the environment [39]. Especially in countries with great social disparity, the relative importance of economic level, security aspects, availability of spaces, and opportunities to practice physical activity in the context of school or neighborhood should be considered. Thus, although studies centered on the linear understanding between motivation and the practice of physical activities are relevant [40, 41], there is still little evidence about the complex relationships between different variables that can explain or mediate the motivation for the practice of physical activity.

In this sense, among the available analysis models, the network analysis can provide a means to understand the relationships at the system level, in order to understand the complex interaction of social and environmental factors that are interconnected with the phenomenon of adolescents’ intention to be physically active to practice physical activity [42]. Therefore, the objective of the study was to explore the contribution of individual characteristics, social and environmental factors - school and region - in the intention to be physically active in Brazilian adolescents.

## Methods

### Design

This is a cross-sectional study using secondary data from the third edition of the National School Health Survey (PeNSE), carried out in Brazil in 2015. The National Adolescent School-based Health Survey (PeNSE) is a survey conducted with adolescent students and is part of the Brazilian Surveillance of Risk and Protective Factors for Chronic Diseases. Its first edition was conducted in 2009 and was planned to be held every three years. Since then, there have been three more editions, one in 2012, one in 2015 and another one in 2019 [43]. The fourth edition of the PeNSE, conducted in 2019, was not used due to the removal of the question “If you had the opportunity to practice physical activity on most days of the week, what would your attitude be?“ which would result in the motivation variable.

The questionnaire was based on the School-Based Student Health Survey [44] which was adapted to the Brazilian context [43]. This survey is the result of a partnership between the Ministry of Health and the Brazilian Institute of Geography and Statistics (IBGE), with support from the Ministry of Education (MEC) [43]. The PeNSE is a health survey that aims to identify risk and protective factors for the health of schoolchildren who study in the day shift. Data were collected from 3,040 schools, 4,159 classes and 102,301 students, composed of two types of samples: sample 1 (students attending the 9th grade of elementary school) and sample 2 (students aged 13 to 17 who are enrolled in primary and secondary education) [45]. The research was approved by the National Research Ethics Committee (CONEP) (Opinion Number. 11.537/2009, 16.805/2012, and 1.006.467/2015).

The samples of 9th graders (Sample 1) were dimensioned so the population parameters (proportions or prevalence) could be estimated, irrespective of geographic locations: each one of the 26 state capitals plus the Federal District was defined as a geographical stratum; the other municipalities were grouped into 26 geographical strata, representing each one of the Brazilian states, including their capitals, totalizing 53 strata. One sample of schools was dimensioned and selected in each one of the 53 strata formed [43].

All students present in the selected classes were invited to participate in the research, but only those who returned with the free and informed consent form duly completed by their legal guardians were included in the sample. More information can be obtained through Oliveira et al. [43].

The study sample included a total of 53,937 adolescents. Only individuals who engaged in less than 300 min of physical activity per week were included.

### Variables and instruments

Data collection was conducted between April and September 2015. For data collection, a self-administered electronic questionnaire was used, in which participants were instructed to consider the last seven days prior to the survey. Data were collected during class hours using smartphones provided by Brazilian Institute of Geography and Statistics. The tool destined to collect data from students was smartphones. The IBGE technician distributed the devices to the students who were in class on the day of the interview and explained to them how to use the device [43]. An application on each device contained a self-administered and structured questionnaire, divided into thematic modules. Students could answer the questionnaire in full or in part. Data were transmitted and processed daily, allowing the smartphones and database to be updated automatically.

Information referring to the school was answered by the director or person responsible for the selected school, providing information including the offer of after-hours sports activities, administrative dependency, and geographic area.

### Intention to be physically active

Participants were asked “If you had the opportunity to practice physical activity most days of the week, what would your attitude be?”. Possible answers were: (1) I wouldn’t practice it anyway; (2) I would practice physical activity on some days of the week; (3) I would practice physical activity most days of the week; (4) I already practice physical activity on some days of the week; (5) I already practice physical activity most days of the week; (99) Not informed. For analysis purposes, only responses 1, 2, and 3 were considered to assess motivation which would or would not lead to the practice of physical activity, disregarding individuals who already practice physical activity.

### Sociodemographic variables

Sociodemographic variables were divided into 4 domains: individual characteristics, social factors, school environment, and regional environment. The individual characteristics were sex (male and female), skin color/race (non-white and white), age (less than 14 years old, 14 years old, and older than 14 years), and body image perception (very thin, thin, normal, fat, and very fat).

Social factors included the perception of safety on the way from home to school or from school to home (safe and unsafe) and maternal education (low education for less than 9 years of schooling, basic education between 9 and 12 years of schooling, and higher education for more than 12 years of schooling). As indicators of the school environment, days of physical education classes were used (no days, up to two days, and more than two days), the offer of after-school sports activities (school does not offer and school does offer), and administrative dependency (public and private). Indicators of the regional environment included the type of municipality (capital and non-capital) and the geographic area (urban or rural). Supplementary box 1 provides a more detailed explanation of how the variables used in this study were collected.

### Statistical procedures

For the characterization of the sample, descriptive statistics were used with the presentation of absolute and relative frequency (%) and respective 95% confidence intervals (95%CI), for the total sample and stratified by level of motivation.

Network analysis was used to estimate the association between motivation and individual characteristics, social and environmental factors (school and region). Supplementary box 2 provides a detailed description of how the variables were used in the network analysis. The “Fruchterman-Reingold” algorithm was applied. Data were shown in relative space where variables with stronger associations remain together and those with less strong associations repel each other. To improve the accuracy of the network, we used the “random Markov fields” model. The algorithm adds an “L1” penalty (regularized neighborhood regression). Regulation is estimated by a less complete selection and contraction operator (Lasso) that controls the sparse network. The Extended Bayesian Information Criterion (EBIC) for selecting the Lambda of the regularization parameter was observed. The EBIC uses a hyperparameter (y) that determines the number of EBIC that select sparse models. The value of y was determined to be 0.50 (range 0 to 0.50). The network analysis uses regularized least absolute shrinkage and selection operator (LASSO) algorithms to obtain the precision matrix (weight matrix). When standardized, this matrix represents the associations between the variables in the network. The network is presented in a graph that includes the variables (nodes) and the relationships (lines). Blue lines represent positive associations and red lines represent negative associations. The thickness and intensity of the lines represent the magnitude of the associations. The centrality indicator was presented through the values of expected influence. The expected influence quantifies the position of the nodes in a network in terms of relative importance Statistical procedures were performed in JASP (version 13.1.0).

## Results

The study included a total of 53,937 adolescents. Only responses 1 (I wouldn’t practice it anyway), 2 (I would practice physical activity on some days of the week) and 3 (I would practice physical activity most days of the week) were considered to evaluate the intention to be physically active, and individuals who engaged in less than 300 min of physical activity per week were included. The majority of adolescents were female (61%), aged 14 years (50%), non-white (67%), considered themselves to be of “normal” regarding their self-image (53%), felt safe on their way to school (89%), had mothers with basic education (40%), participated in up to 2 days of physical education classes (78%), studied in schools that offered after-school physical activity (58%), studied in public schools (81%), were residents in urban areas (91%).

Table 1 shows the distribution of motivation patterns among each population subgroup. The highest proportion of non-motivated students was observed among white female adolescents, over 14 years old, who considered themselves too thin or too fat, who did not feel safe on their way to school, children of mothers with high education, who did not have physical education classes, public school students, and residents of urban areas. The highest proportion of adolescents motivated to practice physical activity on most days of the week was observed in females, aged up to 14 years, who perceived themselves as fat, children of mothers with basic education, who did not attend physical education classes, private school students, and residents of capital cities.

**Table 1:** Distribution of levels of motivation among sociodemographic characteristics in Brazilian adolescents (n = 53,937)

			1			2			3			TOTAL*	
Groups	Variables	N	%	95%CI	N	%	95%CI	N	%	95% CI	N	%	95%CI
		4756	100	---	26,777	100	---	22,404	100		102,072	100	---
**Individual**	**Sex**												
	Male	1834	38.6	(37.2–39.9)	9789	36.6	(36.0–37.1)	9489	42.4	(41.7–43.0)	49,290	48.3	(48.0–48.6)
	Female	2922	61.4	(60.0–62.8)	16,988	63.4	(62.9–64.0)	12,915	57.6	(57.0–58.3)	52,782	51.7	(51.4–52.0)
	**Color/race**												
	Non-white	3005	63.4	(61.8–64.5)	18,203	68.1	(67.4–68.5)	14,871	66.5	(65.8–67.0)	68,189	66.9	(66.5–67.1)
	White	1740	36.6	(35.2–38.0)	8540	31.9	(31.3–32.4)	7511	33.5	(32.9–34.1)	33,775	33.1	(32.8–33.4)
	**Age**												
	14 years old	2274	47.8	(46.4–49.2)	13,389	50.0	(49.4–50.6)	11,570	51.6	(51.0–52.3)	51,611	50.6	(50.3–50.9)
	Older than 14 years old	1737	36.5	(35.2–37.9)	8445	31.5	(31.0–32.1)	6726	30.0	(29.4–30.6)	32,629	32.0	(31.7–32.2)
	Less than 14 years old	745	15.7	(14.6–16.7)	4943	18.4	(18.0–18.9)	4108	18.3	(17.8–18.8)	17,832	17.5	(17.2–17.7)
	**Body image perception**												
	Very thin	482	10.1	(9.3–11.0)	1703	6.3	(6.0–6.7)	1153	5.1	(4.9–5.4)	5369	5.3	(5.1–5.4)
	Thin	1067	22.4	(21.3–23.6)	5968	22.3	(21.8–22.8)	4523	20.2	(19.7–20.7)	20,896	20.5	(20.2–20.7)
	Normal	2266	47.6	(46.2–49.1)	14,431	53.9	(53.3–54.5)	11,591	51.7	(51.1–52.4)	56,611	55.5	(55.2–55.8)
	Fat	674	14.2	(13.2–15.2)	3965	14.8	(14.4–15.2)	4382	19.6	(19.0–20.1)	15,919	15.6	(15.4–15.8)
	Very fat	172	3.6	(3.1–4.2)	457	1.7	(1.6–1.9)	614	2.7	(2.5–3.0)	2200	2.2	(2.1–2.2)
**Social**	**Perception of safety**												
	Safe	4078	85.7	(84.7–86.7)	23,788	88.8	(88.4–89.2)	19,938	89.0	(88.6–89.4)	90,070	88.2	(88.0–88.4)
	Unsafe	617	13.0	(12.0–14.0)	2839	10.6	(10.2–11.0)	2382	10.6	(10.2–11.0)	11,269	11.0	(10.8–11.2)
	**Maternal education**												
	Low education	1089	22.9	(21.7–24.1)	6864	25.6	(25.1–26.2)	5427	24.2	(23.7–24.8)	29,772	29.2	(28.9–29.4)
	Basic education	1224	25.7	(24.5–27.0)	7613	28.4	(27.9–29.0)	7182	32.1	(31.4–32.7)	24,178	23.7	(23.4–23.9)
	High education	938	19.7	(18.6–20.9)	4756	17.8	(17.3–18.2)	4790	21.4	(20.8–21.9)	22,688	22.2	(22.0–22.5)
**School**	**Days of PE classes**												
	No days	1068	22.5	(21.3–23.6)	5009	18.7	(18.2–19.2)	4186	18.7	(18.2–19.2)	16,761	16.4	(16.2–16.6)
	Up to two days	3488	73.3	(72.1–74.6)	20,880	78.0	(77.5–78.5)	17,554	78.3	(77.8–78.9)	73,604	72.1	(71.8–72.4)
	More than two days	185	3.9	(3.4–4.5)	829	3.1	(2.9–3.3)	626	2.8	(2.6–3.0)	11,417	11.2	(11.0–11.4)
	**After-school sports activities**												
	School does not offer	2012	42.4	(40.9–43.7)	11,326	42.4	(41.7–42.9)	9121	40.7	(40.1–41.4)	41,524	40.7	(40.4–41.0)
	School does offer	2741	57.6	(56.2–59.0)	15,417	57.6	(57.0–58.2)	13,256	59.3	(58.5–59.8)	60,407	59.3	(58.9–59.5)
	**Administrative dependency**												
	Public	3966	83.4	(82.3–84.4)	22,078	82.5	(82.0–82.9)	17,405	77.7	(77.1–78.2)	81,154	79.5	(79.3–79.7)
	Private	790	16.6	(15.6–17.7)	4699	17.5	(17.1–18.0)	4999	22.3	(21.8–22.9)	20,918	20.5	(20.2–20.7)
**Regional**	**Type of municipality**												
	Capital	2376	50.0	(48.5–51.4)	13,124	49.0	(48.4–49.6)	11,264	50.3	(49.6–50.9)	51,192	50.1	(49.8–50.5)
	Non-capital	2380	50.0	(48.6–51.4)	13,653	51.0	(50.4–51.6)	11,140	49.7	(49.1–50.4)	50,880	49.9	(49.5–50.1)
	**Geographic area**												
	Urban	4392	92.4	(91.6–93.1)	24,326	90.9	(90.5–91.2)	20,505	91.5	(91.1–91.9)	93,789	91.9	(91.7–92.0)
	Rural	364	7.6	(6.9–8.4)	2451	9.1	(8.8–9.5)	1899	8.5	(8.1–8.8)	8283	8.1	(7.9–8.3)

Note. N = Sample; 1 = I wouldn’t practice it anyway; 2 = I would practice physical activity on some days of the week; 3 = I would practice physical activity most days of the week; 95%CI = 95% confidence interval; PE = Physical education; *Total sample includes also the active adolescents (not included in the main analysis)

**Fig. 1:**
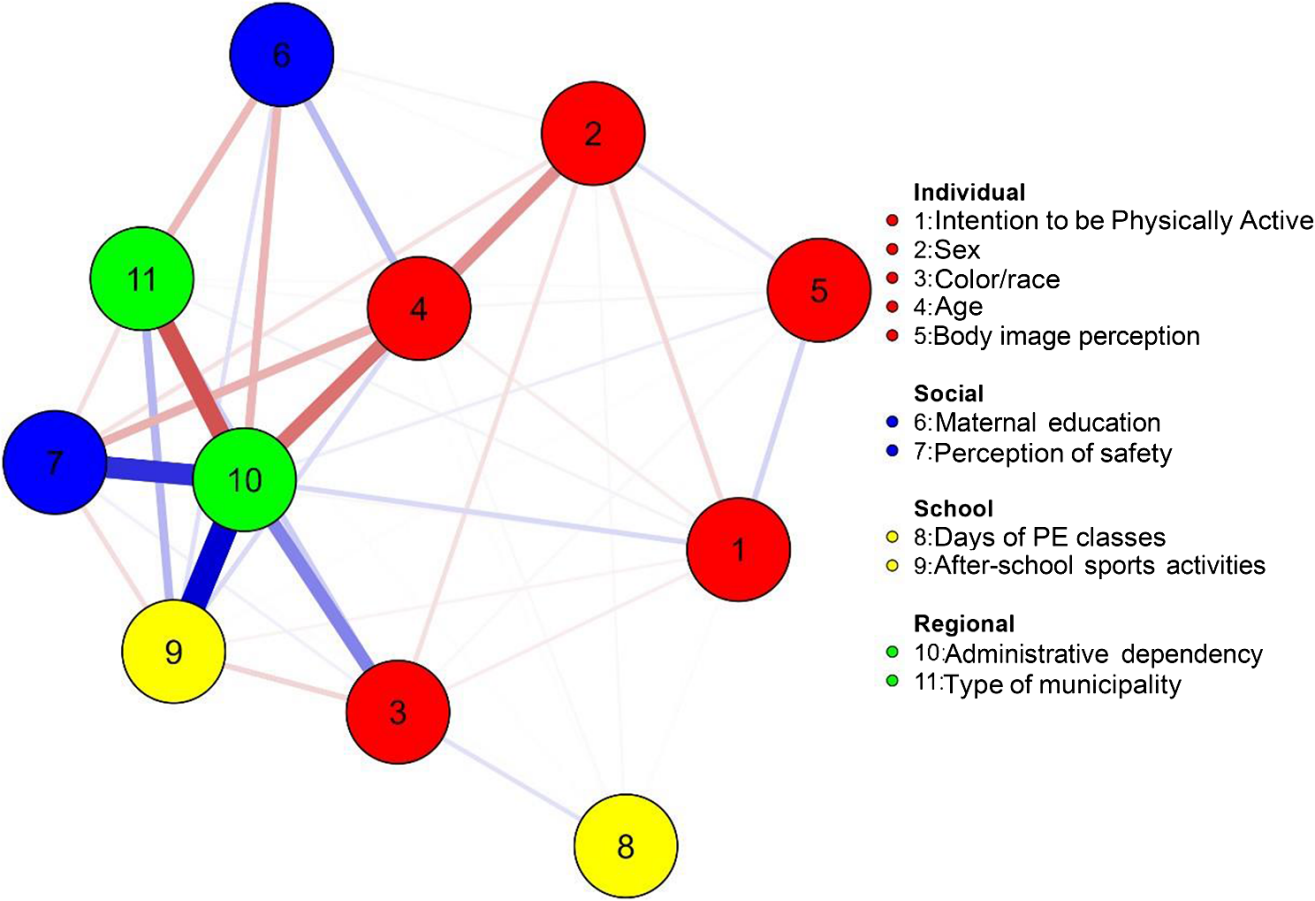
Network topology for the association between motivation and sociodemographic and behavioral variables of Brazilian adolescents (n = 53,937). Note. PE = Physical education

The Fig. 1 presents the results of the network analysis. In association with the weight matrix, the results showed that the intention to be physically active had a negative relationship with sex (-0.073) and a positive relationship with self-image perception (0.079), indicating that boys had higher intentions to be active compared to their peers, as well as adolescents who perceived themselves as fat. Additionally, students from private schools demonstrate a higher intention to regularly engage in physical activities (0.071), and in general, private schools offer more extracurricular physical activities (0.478).

Table 2 presents the results of the network centrality indicator. The higher values for expected influence were shown for after-school sports activities (1.632), administrative dependency (1.244), skin color/race (0.663) and body image perception (0.422). The weight matrix is present in Supplementary Table 1.

**Table 2:** Values of the network centrality indicator (n = 53,937)

Variable	Expected influence
Intention to be Physically Active	-0.093
Sex	-1.333
Skin color/race	0.663
Age	-1.547
Body image perception	0.422
Maternal education	0.146
Perception of safety	-0.374
Days of physical education classes	0.140
After-school sports activities	1.632
Administrative dependency	1.244
Type of municipality	-0.900

## Discussion

Our main results indicate that among individual characteristics, social and environmental factors - from the school and the region – individual and environmental variables were more strongly related to the intention to be active in the network. Thus, Brazilian adolescent boys who perceived themselves as fat showed greater intentions to be active. Additionally, students from private schools show a higher intention to regularly engage in physical activities, and in general, private schools offer more extracurricular physical activities.

Previous studies have mainly focused on the role of the teacher in motivating their students for physical education classes and for physical activity outside of school, without considering the multifactorial nature of the phenomenon [30]. On the other hand, there are studies that indicate that autonomous motivation for physical activity is based on the social context and an environment that supports autonomy, providing support or hindering its development [46–49]. The socioeconomic context influences adolescent behavior and is therefore part of the psychosocial process from childhood to adulthood [50]. The lack of information about students’ and their families’ socioeconomic context may contribute to inaccuracies when analyzing the intention to be physically active. Because of this, previous studies have identified the inclusion of these aspects as a gap in the literature [51]. Also, previous studies usually investigated isolated variables, using linear models to examine the relationship between health behaviors and the social and environmental context [52]. Although these studies provide important information about the intention to be physically active and the social context, network analysis can be an important tool to investigate the complex interaction of individual, social, and environmental, school, and regional factors that stand out in the context of motivated behavior. To our knowledge, this is the first national sample study that used a network perspective. Network analysis, as a way of measuring complex systems, provides a topological structure of a network that can be useful for understanding nonlinear relationships and is highly sensitive. In addition, the role of each variable in the network can be better understood from the centrality measures used [42].

Our results showed that adolescents’ intention to be physically active was associated with sex, indicating that boys showed higher intentions to be active. This is a result that agrees with the literature. Previous studies have also pointed to women’s lower autonomous motivation for physical activity [24, 40]. This fact may be related to the perception that physical activities are predominantly masculine, and that competitive sports and school physical education are discouraging and unpleasant due to the emphasis on skill [45]. Additionally, there is pressure to perform well in team sports and fear of criticism and embarrassment, especially in the presence of men with negative attitudes towards female participation in exercise [53, 54]. Therefore, unstructured but organized activities, and non-competitive activities and classes that promote a positive learning environment seem to encourage young girls to maintain higher levels of physical activity [43].

The physical education environment can be stressful for students who face body image problems or have less sports skills, as their bodies are exposed to social comparisons and public performance evaluations [9]. Our study also showed that girls perceive themselves as more fat, suggesting that the “effect” of body image on motivation may be stronger for adolescent girls compared to boys [55]. There is also evidence that improved positive body image is associated with autonomous physical activity motivation [56]. Our study identified a positive relationship between body image and the intention to be physically active, showing that adolescents who perceive themselves as fat have greater intentions to be physically active. However, the intention to be physically active was not significantly related to social variables.

The type of school is one of the most prominent variables regarding the centrality values of the network. Furthermore, from the network topology, it is apparent that students from private schools have a higher intention to be physically active. Additionally, private schools offer more extracurricular physical activities. This may suggest a relationship between the intention to be physically active and socioeconomic status, considering that in Brazil, enrollment in private schools is strongly related to economic level. Our results did not show significant associations with the number of physical education classes and the intention to be active in the network analysis. While physical education classes have proven to be a conducive environment for promoting physical activity [57], our data confirm that the responsibility for promoting physical activity among children and adolescents should be shared by the entire school environment. Strategies that involve physical activities in the classroom, such as breaking up class time with active breaks, incorporating physical activity into the delivery of academic content, and restructuring the classroom to increase physical activity or reduce sedentary behavior, can have a positive impact on the intention to be physically active, levels of physical activity, and academic performance [58]. Moreover, adolescence is a crucial period, linked to a series of physical, social, and cognitive changes. These changes often generate certain feelings of rejection or shyness in different situations that occur during physical education classes, which hinder or inhibit students from performing a particular motor task [59].

One of the factors that impact an active lifestyle during adolescence is physical development, which tends to lead them to compare themselves with their peers. Additionally, social relationships in adolescence are more intense than in childhood, which increases the search for a sense of belonging to a group of friends and can cause them to avoid situations that may harm their social image [60]. Furthermore, students’ motivation for physical education classes can help increase young people’s leisure physical activity, despite any negative experiences they may have had [61]. Physical education classes with teaching that supports autonomy can provide opportunities for students to experience successful physical practices and improve their confidence and motor skills. Teacher support for autonomy and increased autonomous motivation in physical education can also help adolescents perceive greater acceptance of others’ bodies and shift their focus from body appearance to physical activity [62].

Our results have clear practical implications. Firstly, there is a need to explore the specific determinants of physical activity and promote physical activity opportunities for girls and adolescents with poor body image, as these are groups more likely to participate in physical activity programs. Additionally, our results showed that simply offering more physical education classes and physical activity opportunities through school is not enough to motivate students to be active. Physical education teachers are recommended to use pedagogical strategies that promote motivated behavior for physical activity. This can be an effective strategy for promoting physical self-perception and positive body image in female adolescents, especially those with weak physical activity habits. In addition, based on the leverage points identified [63], we also highlight the potential role of after-school sports activities for interventions to impact in the whole system, considering this was the factor with the highest expected influence in the network.

This study has limitations that need to be mentioned. Firstly, being a cross-sectional study, it is not possible to assume causality between the variables and it is possible that the network topology and key variables for understanding the system may change over time. Also, not all the components of the system could be analyzed, and further studies are warranted to better understanding of the system. Additionally, data on motivation, attendance in physical education classes, and physical activity were obtained through a self-reported questionnaire. While questionnaires do not provide the same level of objectivity as accelerometry [64], they are adequate tools to indicate the domain of physical activity that was performed and have a more accessible operational cost, facilitating research with large samples [64]. However, as strengths of this study we highlighted the theoretical framework used to guide the research question, the choice of variables, the representativeness of the data within the Brazilian context, and the statistical analysis that allows addressing the multifactorial characteristics of the studied phenomenon, as well as providing practical suggestions based on the main findings. Network analysis enables the evaluation of non-linear and complex interactions among variables of different natures, which is a limitation of linear models such as regression and structural equations. In network analysis, even variables with few connections can be retained in the model. Additionally, centrality measures like expected influence can be useful to identify variables that could be targets for interventions. This way of understanding the role of each variable is unique to network analysis.

## Conclusions

In conclusion, individual factors such as sex and body image perception, and environmental factors such as school administrative dependency and the availability of after-school activities had a significant contribution to the intention to be physically active among Brazilian adolescents. Given that the intention to be physically active is key to starting and maintaining behaviors that are beneficial to health, motivational strategies to increase participation in physical activity can be important tools for contributing to the overall development of adolescents.

### Electronic supplementary material

Below is the link to the electronic supplementary material.

Supplementary Material 1

Supplementary Material 2

Supplementary Material 3

## Data Availability

The data presented in this study are openly available in https://www.ibge.gov.br/estatisticas/downloads-estatisticas.html?caminho=pense/2015/microdados/.
