# Supplementary material for: What are the correlates of intention to be physically active in Brazilian adolescents? A network analysis
Source: BMC Public Health. 2023 Dec 8;23:2460. doi: 10.1186/s12889-023-17291-2 (PMC10709911; doi:10.1186/s12889-023-17291-2)
Supplement: Supplementary file 1 — Supplementary Material 1 [file 12889_2023_17291_MOESM1_ESM.docx]

**Supplementary table 1.** Weights matrix

|  | | | | | | | | | | | | | | | | | | | | | | | | | |
| --- | --- | --- | --- | --- | --- | --- | --- | --- | --- | --- | --- | --- | --- | --- | --- | --- | --- | --- | --- | --- | --- | --- | --- | --- | --- |
|  | | **Network** | | | | | | | | | | | | | | | | | | | | | | | |
| **Variable** | | **1** | | **2** | | **3** | | **4** | | **5** | | **6** | | **7** | | **8** | | **9** | | **10** | | | **11** | | |
| **Intention to be Physically Active (1)** |  | 0.000 |  | -0.073 |  | -0.033 |  | -0.035 |  | 0.079 |  | -0.001 |  | -0.013 |  | 0.006 |  | -0.023 |  | 0.071 |  | 0.021 | |  |  |
| **Sex (2)** |  | -0.073 |  | 0.000 |  | -0.059 |  | -0.209 |  | 0.054 |  | -0.019 |  | -0.057 |  | -0.013 |  | 0.000 |  | -0.034 |  | -0.001 | |  |  |
| **Skin color/race (3)** |  | -0.033 |  | -0.059 |  | 0.000 |  | -0.002 |  | 0.014 |  | 0.000 |  | 0.027 |  | 0.057 |  | -0.078 |  | 0.232 |  | 0.094 | |  |  |
| **Age (4)** |  | -0.035 |  | -0.209 |  | -0.002 |  | 0.000 |  | -0.020 |  | 0.125 |  | -0.147 |  | 0.010 |  | 0.069 |  | -0.265 |  | -0.011 | |  |  |
| **Perception of self-image (5)** |  | 0.079 |  | 0.054 |  | 0.014 |  | -0.020 |  | 0.000 |  | 0.005 |  | 0.002 |  | 0.002 |  | -0.006 |  | 0.032 |  | 0.008 | |  |  |
| **Perception of safety (6)** |  | -0.001 |  | -0.019 |  | 0.000 |  | 0.125 |  | 0.005 |  | 0.000 |  | 0.000 |  | 0.005 |  | 0.059 |  | -0.137 |  | -0.131 | |  |  |
| **Maternal education (7)** |  | -0.013 |  | -0.057 |  | 0.027 |  | -0.147 |  | 0.002 |  | 0.000 |  | 0.000 |  | 0.004 |  | -0.066 |  | 0.391 |  | -0.063 | |  |  |
| **Days of PE classes (8)** |  | 0.006 |  | -0.013 |  | 0.057 |  | 0.010 |  | 0.002 |  | 0.005 |  | 0.004 |  | 0.000 |  | 0.002 |  | 0.000 |  | 0.003 | |  |  |
| **Offer of after-school sports activities (9)** |  | -0.023 |  | 0.000 |  | -0.078 |  | 0.069 |  | -0.006 |  | 0.059 |  | -0.066 |  | 0.002 |  | 0.000 |  | 0.478 |  | 0.136 | |  |  |
| **Administrative dependency (10)** |  | 0.071 |  | -0.034 |  | 0.232 |  | -0.265 |  | 0.032 |  | -0.137 |  | 0.391 |  | 0.000 |  | 0.478 |  | 0.000 |  | -0.325 | |  |  |
| **Type of municipality (11)** |  | 0.021 |  | -0.001 |  | 0.094 |  | -0.011 |  | 0.008 |  | -0.131 |  | -0.063 |  | 0.003 |  | 0.136 |  | -0.325 |  | 0.000 | |  |  |
|  | | | | | | | | | | | | | | | | | | | | | | | | | |

Note. PE = Physical Education.
