# Supplementary material for: What are the correlates of intention to be physically active in Brazilian adolescents? A network analysis
Source: BMC Public Health. 2023 Dec 8;23:2460. doi: 10.1186/s12889-023-17291-2 (PMC10709911; doi:10.1186/s12889-023-17291-2)
Supplement: Supplementary file 2 — Supplementary Material 2 [file 12889_2023_17291_MOESM2_ESM.docx]

**Supplementary box 1.** Characterization of the variables used.

| **Groups** | **Variable** | **Question used** | **Possible response options in PeNSE** | **Classification for the study** |
| --- | --- | --- | --- | --- |
| **Individual** | **Intention to be Physically Active** | **If you had the opportunity to practice physical activity most days of the week, what would your attitude be?** | I wouldn't practice it anyway | I wouldn't practice it anyway |
|  |  |  | I would practice physical activity on some days of the week |  |
|  |  |  | I would practice physical activity most days of the week | I would practice physical activity on some days of the week |
|  |  |  | I already practice physical activity on some days of the week |  |
|  |  |  | I already practice physical activity most days of the week | I would practice physical activity most days of the week |
|  |  |  | Not informed |  |
|  | **Sex** | **What is your sex?** | Male | Male |
|  |  |  | Female | Female |
|  | **Color/race** | **What color or race are you?** | Black | Non-white |
|  |  |  | Yellow |  |
|  |  |  | Brown |  |
|  |  |  | Indigenous |  |
|  |  |  | White | White |
|  |  |  | Not informed |  |
|  | **Age** | **What is your age?** | 11 years old or younger | Ascending order from 11 to 18 years old |
|  |  |  | 12 years old |  |
|  |  |  | 13 years old |  |
|  |  |  | 14 years old |  |
|  |  |  | 15 years old |  |
|  |  |  | 16 years old |  |
|  |  |  | 17 years old |  |
|  |  |  | 18 years old |  |
|  |  |  | 19 years old or older |  |
|  | **Perception of self-image** | **Regarding your body, do you consider yourself:** | Very thin | Very thin |
|  |  |  | Thin | Thin |
|  |  |  | Normal | Normal |
|  |  |  | Fat | Fat |
|  |  |  | Very fat | Very fat |
| **Social** | **Perception of safety** | **In the last 30 days, on how many days did you skip school because you didn't feel safe on the way from home to school or from school to home?** | No days in the last 30 days (0 days) | Safe |
|  |  |  | 1 day in the last 30 days | Unsafe |
|  |  |  | 2 days in the last 30 days |  |
|  |  |  | 3 days in the last 30 days |  |
|  |  |  | 4 days in the last 30 days |  |
|  |  |  | 5 days or more in the last 30 days |  |
|  |  |  | Not informed |  |
|  | **Maternal education** | **What level of education (grade) did your mother study or is currently studying?** | My mother did not study. | Low education |
|  |  |  | My mother started elementary school or 1st grade but did not finish. |  |
|  |  |  | My mother completed elementary school or 1st grade. |  |
|  |  |  | My mother started high school or 2nd grade but did not finish. | Basic education |
|  |  |  | My mother completed high school or 2nd grade. |  |
|  |  |  | My mother started college (higher education) but did not finish. | High education |
|  |  |  | My mother completed college (higher education). |  |
|  |  |  | I don't know. |  |
|  |  |  | Not informed |  |
| **School** | **Days of PE classes** | **In the last 7 days, how many days did you have physical education classes at school?** | No days in the last 7 days (0 days) | No days |
|  |  |  | 1 day in the last 7 days | Up to two days |
|  |  |  | 2 days in the last 7 days |  |
|  |  |  | 3 days in the last 7 days | More than two days |
|  |  |  | 4 days in the last 7 days |  |
|  |  |  | 5 days in the last 7 days |  |
|  |  |  | 5 days plus Saturday in the last 7 days |  |
|  |  |  | 5 days plus Saturday and Sunday in the last 7 days |  |
|  |  |  | Not informed |  |
|  | **After-school sports activities** | **Does the school offer sports activities for students outside regular class hours?** | Data not available. | School does not offer |
|  |  |  | No. |  |
|  |  |  | Yes, free. | School does offer |
|  |  |  | Yes, paid. |  |
|  |  |  | Yes, paid and free. |  |
|  |  |  | Not informed |  |
|  | **Administrative dependency** | **Administrative Dependency of the school.** | Public | Public |
|  |  |  | Private | Private |
| **Regional** | **Type of municipality** | **Capital municipality indicator.** | Capital | Capital |
|  |  |  | Non-capital | Non-capital |
|  | **Geographic area** | **School situation** | Urban | Urban |
|  |  |  | Rural | Rural |

Note. PE = Physical Education.
