# Supplementary material for: What are the correlates of intention to be physically active in Brazilian adolescents? A network analysis
Source: BMC Public Health. 2023 Dec 8;23:2460. doi: 10.1186/s12889-023-17291-2 (PMC10709911; doi:10.1186/s12889-023-17291-2)
Supplement: Supplementary file 3 — Supplementary Material 3 [file 12889_2023_17291_MOESM3_ESM.docx]

**Supplementary box 2.** Coding in network analysis.

| **Groups** | **Variables** | **Coding in network analysis** |
| --- | --- | --- |
| **Individual** | **Intention to be Physically Active** |  |
|  | I wouldn't practice it anyway | 1 |
|  | I would practice physical activity on some days of the week | 2 |
|  | I would practice physical activity most days of the week | 3 |
|  | **Sex** |  |
|  | Male | 1 |
|  | Female | 2 |
|  | **Color/race** |  |
|  | Non-white | 1 |
|  | White | 2 |
|  | **Age** |  |
|  | Ascending order from 11 to 18 years old | |
|  | **Perception of self-image** |  |
|  | Very thin | 1 |
|  | Thin | 2 |
|  | Normal | 3 |
|  | Fat | 4 |
|  | Very fat | 5 |
| **Social** | **Perception of safety** |  |
|  | Safe | 1 |
|  | Unsafe | 2, 3, 4, 5 and 6 |
|  | **Maternal education** |  |
|  | Low education | 1 and 2 |
|  | Basic education | 3, 4 and 5 |
|  | High education | 6 and 7 |
| **School** | **Days of PE classes** |  |
|  | No days | 0 |
|  | Up to two days | 1 |
|  | More than two days | 2 |
|  | **After-school sports activities** |  |
|  | School does not offer | 1 |
|  | School does offer | 2 |
|  | **Administrative dependency** |  |
|  | Public | 1 |
|  | Private | 2 |
| **Regional** | **Type of municipality** |  |
|  | Capital | 1 |
|  | Non-capital | 2 |
|  | **Geographic area** |  |
|  | Urban | 1 |
|  | Rural | 2 |

Note. PE = Physical Education.
